# Supplementary material for: The interactome of intact mitochondria by cross-linking mass spectrometry provides evidence for coexisting respiratory supercomplexes
Source: Mol Cell Proteomics. 2017 Dec 8;17(2):216–32. doi: 10.1074/mcp.RA117.000470 (PMC5795388; doi:10.1074/mcp.RA117.000470)
Supplement: Supplemental Data [file supp_RA117.000470_133922_0_supp_23978_fzffwf.zip › spectra_annotation/mito_spectra_annotation/436-1-7-1-24-1.pdf]

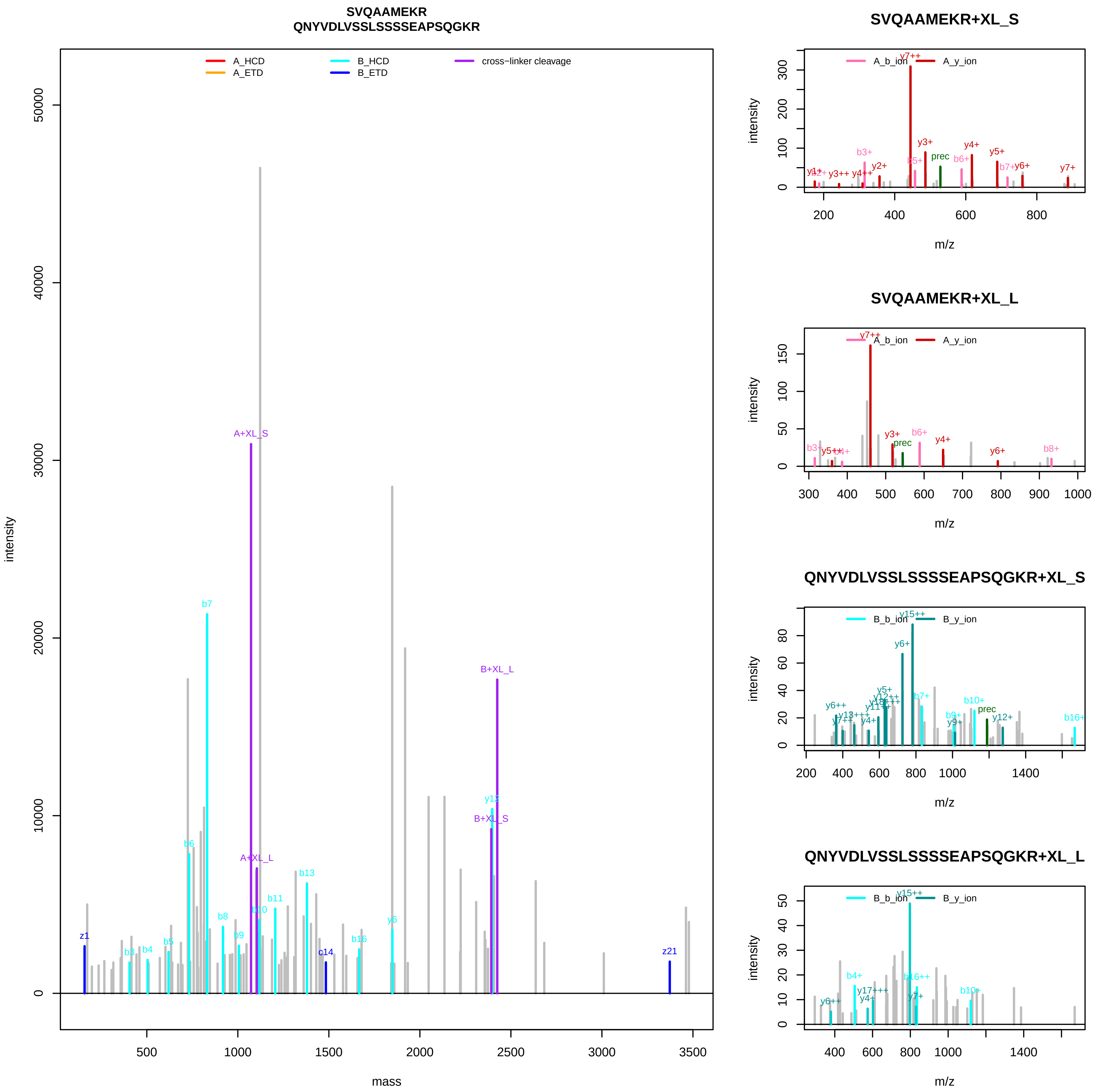
SVQAAMEKR
QNYVDLVSSLSSSSEAPSQGKR
A_HCD
A_ETD
B_HCD
B_ETD
cross−linker cleavage
A+XL_S
A+XL_L
B+XL_L
B+XL_S
z1
b3
b4
b5
b6
b7
b8
b9
b10
b11
b13
c14
b16
y6
y12
z21
intensity
mass
0
10000
20000
30000
40000
50000
500
1000
1500
2000
2500
3000
3500
SVQAAMEKR+XL_S
A_b_ion
A_y_ion
y7++
y3+
prec
y4+
y5+
b3+
b5+
b6+
b7+
y6+
y7+
y1+
b2+
y3++
y4++
y2+
intensity
m/z
0
100
200
300
200
400
600
800
SVQAAMEKR+XL_L
A_b_ion
A_y_ion
y7++
b3+
y5++
b4+
y3+
prec
b6+
y4+
y6+
b8+
intensity
m/z
0
50
100
150
300
400
500
600
700
800
900
1000
QNYVDLVSSLSSSSEAPSQGKR+XL_S
B_b_ion
B_y_ion
y15++
y6+
y5+
y12++
y18++
y11++
y6++
y7++
y13+++
y4+
b7+
b10+
prec
b9+
y9+
y12+
b16+
intensity
m/z
0
20
40
60
80
200
400
600
800
1000
1400
QNYVDLVSSLSSSSEAPSQGKR+XL_L
B_b_ion
B_y_ion
y15++
b4+
y17+++
y4+
y6++
b16++
y7+
b10+
intensity
m/z
0
10
20
30
40
50
400
600
800
1000
1400
